# Thyroid Cancer and COVID-19: Prospects for Therapeutic Approaches and Drug Development

**DOI:** 10.3389/fendo.2022.873027

**Published:** 2022-05-04

**Authors:** Na Qu, Zongguang Hui, Zhixin Shen, Chengxia Kan, Ningning Hou, Xiaodong Sun, Fang Han

**Affiliations:** ^1^ Department of Endocrinology and Metabolism, Affiliated Hospital of Weifang Medical University, Weifang, China; ^2^ Clinical Research Center, Affiliated Hospital of Weifang Medical University, Weifang, China; ^3^ Department of Pathology, Affiliated Hospital of Weifang Medical University, Weifang, China; ^4^ Department of Breast and Thyroid Surgery, Affiliated Hospital of Weifang Medical University, Weifang, China

**Keywords:** thyroid cancer, COVID-19, immunotherapy, multikinase inhibitors, drug target

## Abstract

Thyroid cancer is the most prevalent endocrine malignancy and the reported incidence of thyroid cancer has continued to increase in recent years. Since 2019, coronavirus disease 2019 (COVID-19) has been spreading worldwide in a global pandemic. COVID-19 aggravates primary illnesses and affects disease management; relevant changes include delayed diagnosis and treatment. The thyroid is an endocrine organ that is susceptible to autoimmune attack; thus, thyroid cancer after COVID-19 has gradually attracted attention. Whether COVID-19 affects the diagnosis and treatment of thyroid cancer has also attracted the attention of many researchers. This review examines the literature regarding the influence of COVID-19 on the pathogenesis, diagnosis, and treatment of thyroid cancer; it also focuses on drug therapies to promote research into strategies for improving therapy and management in thyroid cancer patients with COVID-19.

## Introduction

Thyroid cancer is the most prevalent endocrine malignancy worldwide (1). The reported incidence of thyroid cancer is continually increasing because of advanced diagnostic imaging and frequent surveillance (2). According to tumor origin and differentiation characteristics, thyroid cancer is classified as differentiated thyroid cancer (DTC), medullary thyroid carcinoma (MTC), and anaplastic thyroid cancer (ATC). DTC [including papillary thyroid carcinoma (PTC) and follicular thyroid carcinoma] constitutes approximately 85%–95% of thyroid cancers (3). Patients with thyroid cancer often experience hoarseness, dysphagia, and dyspnea; they have a large, poorly mobile, painless mass that can be palpated in the thyroid area. Thyroid cancer metastases most often occur in the lymph nodes of the neck, followed by the lungs, brain, and bones (4). Notably, PTC has a better prognosis with reduced mortality. However, other types of thyroid cancer (e.g., ATC) have a worse prognosis (5). The main goal of thyroid cancer treatment is to improve both overall survival and quality of life. Nevertheless, the main challenges remain improving the rate of early diagnosis and choosing the most appropriate treatment; there is also a need to establish basic requirements for reducing unnecessary injury and improving the quality of life.

In December 2019, the severe acute respiratory syndrome coronavirus 2 (SARS-CoV-2) began to spread worldwide; it has caused the coronavirus disease 2019 (COVID-19) global pandemic (6). The main features of COVID-19 include fever, fatigue, occasional headaches, and other influenza-like symptoms. While 5% of patients exhibit pneumonia or other severe illnesses, most patients only experience mild symptoms and have a good prognosis (7). Currently, most treatment is symptomatic and supportive because no specific treatment has been proven effective. Importantly, with increasing understanding of the disease and global vaccination efforts, the rates of COVID-19 morbidity and mortality have declined (8). Nevertheless, patients with chronic diseases (e.g., cancer) carry risks of additional complications. COVID-19 aggravates primary illnesses and affects disease management; relevant changes include delayed diagnosis and treatment (9). COVID-19 pathogenesis involves features of autoimmunity (10). The entry of SARS-CoV-2 into patients can induce a cytokine storm, which promotes viral entry and accelerates immune evasion (11). The thyroid is an endocrine organ that is susceptible to autoimmune attack; thus, thyroid cancer after SARS-CoV-2 infection has gradually attracted attention. This review focuses on clinical therapies to promote research into strategies for improving therapy and management in thyroid cancer patients with COVID-19.

## Mechanisms of Pathogenesis in Thyroid Cancer and COVID-19

### The Pathogenesis of Thyroid Cancer

**Figure 1:**
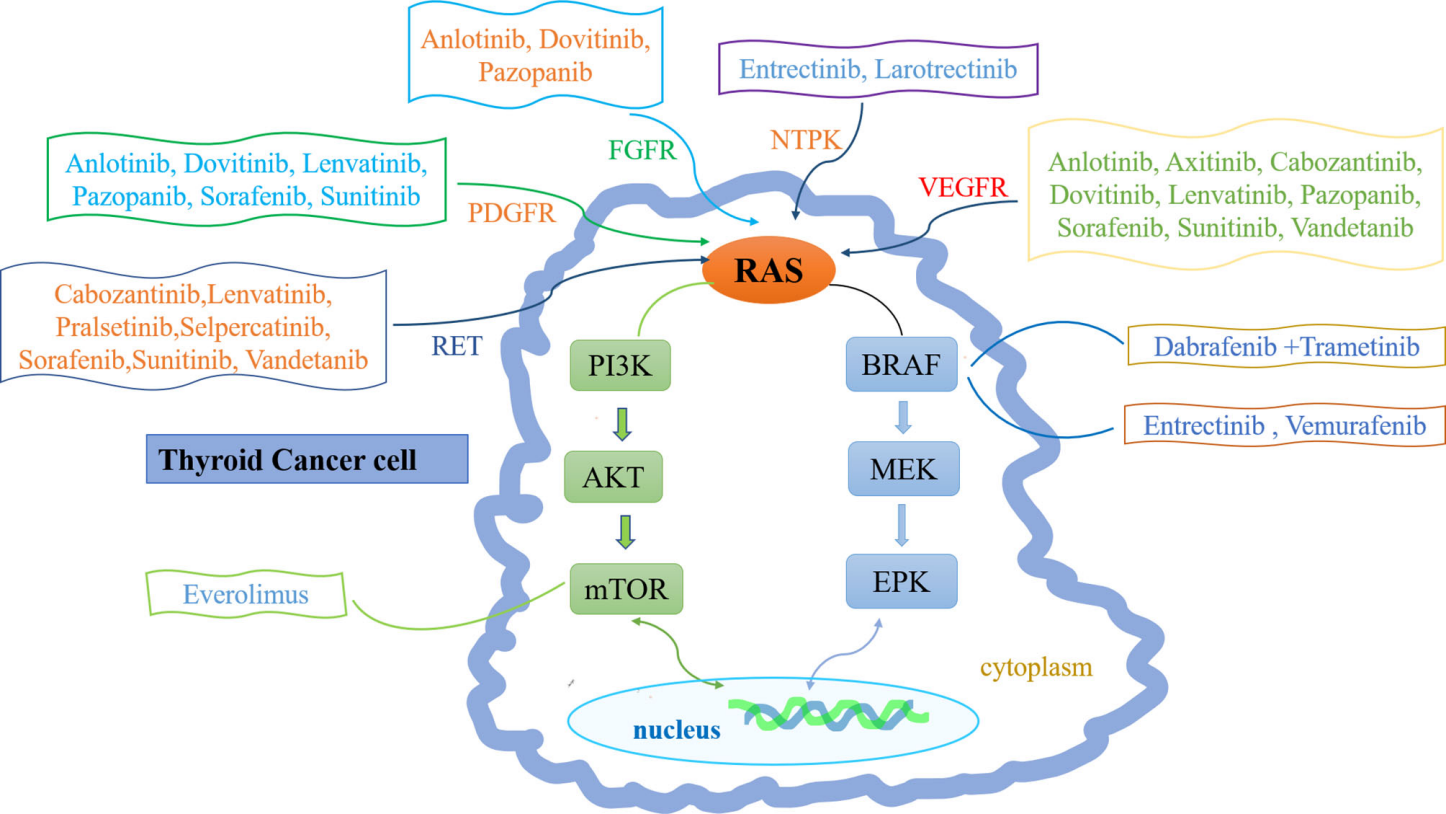
Molecular pathogenesis and therapeutic drug targets of thyroid cancer. The molecular pathogenesis of thyroid cancer mainly involves the imbalance of two classical signaling pathways: mitogen-activated protein kinase (MAPK) (right) and phosphatidylinositol-3 kinase (PI3K)/Akt (left). Ras can transmit receptor tyrosine kinase (RTK) signals to these two pathways. Common mutations in MAPK pathway include RET-PTC and NTPK rearrangement, RAS and BRAF mutations. Common changes in PI3K pathway include RAS mutation, PTEN mutation or deletion, PIK3CA mutation or amplification and AKT1 mutation. Inhibitor drugs that block these signals are also depicted.

The pathogenesis of thyroid cancer includes gene mutation, rearrangement and fusion, abnormal gene expression, and signaling pathway abnormalities (3). Most MTCs are sporadic and involve somatic rearrangement during transfection (RET) mutations. Only 25% of MTCs are hereditary and involve germline RET mutations (12). Mutations also occur in ATC; the main mutation sites are in TP53, BRAF, and NRAS genes, but the incidences of such mutations are lower in ATC than in DTC (13). Gene rearrangement and fusion commonly involve BRAF, RET, NTRK1, NTRK3, and fibroblast growth factor (14). Abnormal gene expression patterns (e.g., involving vascular endothelial growth factor [VEGF]) can also lead to DTC progression (15). Signaling pathway abnormalities include overactivation of the phosphoinositide 3-kinase/Akt/mechanistic target of rapamycin (mTOR) and WNT/β-catenin pathways (16, 17) **(** **Figure 1** **)**.

### Comorbid COVID-19 and Thyroid Cancer

**Figure 2:**
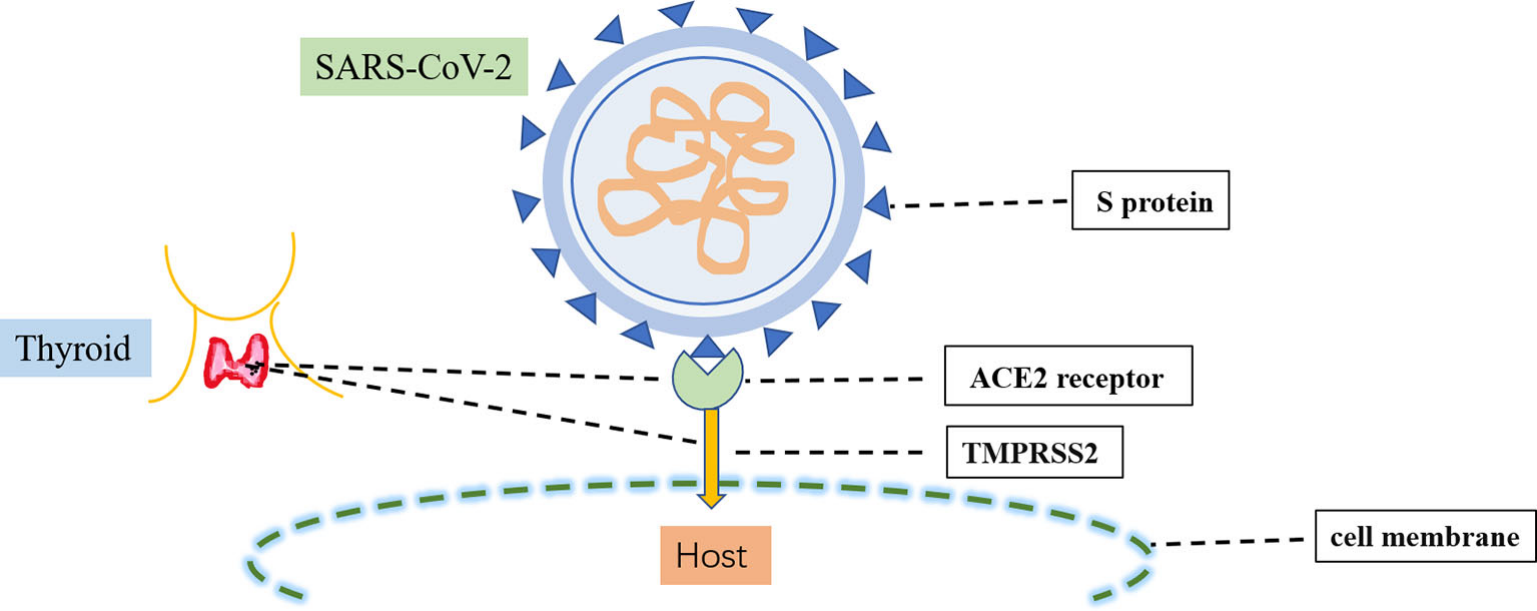
Infection process of SARS-CoV-2. SARS-CoV-2 infection involves two crucial steps: the initial recognition of the receptor angiotensin-converting enzyme 2 (ACE2) *via* S protein and then the effective fusion of the cell membrane *via* transmembrane protease serine 2 (TMPRSS2). Both ACE2 and TMPRSS22 are highly expressed in the thyroid.

The causative pathogen of COVID-19, SARS-CoV-2, is a single-stranded RNA β-coronavirus 2 with a length of 29.9 kb (18). Two main proteins expressed by SARS-CoV-2 have critical roles in the manifestations of COVID-19 **(** **Figure 2** **)**. The first protein is the SARS-CoV-2 main protease, conserved among coronaviruses with no human homolog; it is mainly responsible for viral transcription and replication (19). The second protein is the spike protein on the outer surface of SARS-CoV-2 viral particles; it is responsible for transmission of SARS-CoV-2 infection (20). There are two crucial steps in SARS-CoV-2 infection: initial recognition of the receptor angiotensin-converting enzyme 2 *via* S protein and effective fusion of the cell membrane *via* transmembrane protease serine 2 (21). Both angiotensin-converting enzyme 2 and transmembrane protease serine 2 are highly expressed in several endocrine tissues (e.g., thyroid, testicular, and adipose tissues) (22). These endocrine tissues may be susceptible to viral attack. SARS-CoV-2 RNA has been detected in the thyroid, implying that SARS-CoV-2 can infect the thyroid and alter the pituitary–thyroid axis (23).

SARS-CoV-2 infection has been reported to induce thyroid disease, including subacute thyroiditis, Hashimoto’s thyroiditis, thyrotoxicosis with thyroid dysfunction, and severe low triiodothyronine syndrome (24, 25). SARS-CoV-2 appears to influence thyroid function through multiple mechanisms. Thyroxine may enhance the internalization of SARS-CoV-2 and worsen the prognosis of COVID-19. Furthermore, SARS-CoV-2 may induce an aggressive inflammatory response and cytokine storm, causing damage to the airway and thyroid (26). When the respiratory system experiences SARS-CoV-2 infection that triggers a cytokine storm, the thyroid is affected, particularly in patients with thyroid cancer (23). Additionally, the thyroid has direct effects on immunity because thyroid hormones serve as modulators of the immune response (27). The thyroid engages in complex interactions with viral infections through hormonal and immunoregulatory signaling molecules (28). Tumor cells usually induce immune system dysfunction, characterized by impaired T cell-mediated cytotoxicity and decreased T-cell proliferation. These changes make cancer patients more susceptible to SARS-CoV-2 infection (29). Thus, the severity of COVID-19 depends on inflammatory and immune responses to SARS-CoV-2 (10) **(** **Table 1** **)**.

**Table 1:** Correlation between COVID-19 and thyroid cancer.

	Relationship to COVID-19	Relationship to thyroid cancer
SARS-CoV-2	the causative agent of COVID-19	SARS-CoV-2 RNA detected in thyroid
ACE2	the cell membrane receptor of SARS-COV-2, preliminary identification by S protein	high expression in thyroid tissue, low expression in thyroid cancer
TMPRSS2	activates the interaction of the S protein of SARS-CoV-2 with ACE2, fuses with the cell membrane, and enters the host cell	highly expressed in thyroid tissue
cytokine storm	caused by COVID-19 infection, accelerated immune evasion	thyroid is vulnerable to viral and immune attack, especially in patients with thyroid cancer

ACE2, angiotensin-converting enzyme 2; COVID-19, coronavirus disease 2019; SARS-CoV-2, severe acute respiratory syndrome coronavirus 2; TMPRSS2, transmembrane protease serine 2.

## Strategies for Surveillance and Diagnosis During the COVID-19 Pandemic

Patients with thyroid cancer usually have no symptoms and occasionally find nodules during physical examinations. Cervical ultrasound and fine-needle aspiration are regarded as first-line methods for thyroid nodule evaluation, particularly in patients with thyroid microcarcinoma (30). Regular follow-up visits are essential for nodule alteration monitoring and the prevention of thyroid cancer recurrence.

During the COVID-19 pandemic, there is a need to re-assess the protocols for surveillance and diagnosis of thyroid cancer. The procedures for diagnosis of thyroid nodules can be safely postponed (31); a few months of diagnostic delay during the COVID-19 pandemic has not negatively impacted most thyroid cancer patients or led to worse disease outcomes (32). However, patients with suspected MTC, ATC, or metastatic thyroid cancer (or a history of one or more of these diseases) should not delay fine-needle aspiration (31). There is an important infectious disease risk associated with fine-needle aspiration: the collection and fixation steps may generate aerosols and droplets that spread viral particles. Therefore, medical staff should wear personal protective masks with filter respirators and face masks to protect their eyes and fix cytology specimens with ethanol when smearing (33).

Compared with non-cancer patients, cancer patients are more likely to experience multiple organ damage after SARS-CoV-2 infection (34). Regardless of active cancer status or treatment status, the risks of death and severe illness because of SARS-CoV-2 infections are increased in cancer patients (35, 36). Therefore, the interaction between thyroid and COVID-19 is complex and bidirectional, further research and analysis are needed (37).

## Therapeutic Approaches During the COVID-19 Pandemic

### Surgical Resection

Treatment of thyroid cancer includes surgical resection, radioiodine therapy (RAI), and pharmacotherapy. Surgical resection is often the first-line treatment approach. Total thyroidectomy is recommended for most DTCs, MTCs, macroscopic metastatic invasive PTCs, and follicular thyroid carcinomas with vascular infiltration (38). Most patients with ATC cannot undergo surgery. If the tumor is excessively large or compresses the trachea (thus causing dyspnea), tumor reduction surgery and tracheotomy can be performed to alleviate the associated symptoms.

The spread of COVID-19 has brought unprecedented challenges to the surgical resection treatment of thyroid cancer. Both diagnostic and therapeutic procedures expose patients to the risk of COVID-19 infection. Moreover, conventional follow-up visits have been postponed in relation to the threat of COVID-19 infection. Because of social distancing restrictions and operating room shortages, all procedures that may nebulize breathing and digest secretions (e.g., endoscopy, tracheal intubation, non-invasive ventilation, and endoscopic surgery) are restricted because they will increase the risk of infection (39). Thus, during the COVID-19 pandemic, the selection of surgical treatment is more cautious (40). The choice to continue or postpone surgery remains controversial because some aspects of disease progression have not been fully elucidated. All surgical options should be carefully reviewed, and plans should be formulated to minimize the risks.

Most DTCs and MTCs are slow-growing tumors; surgical treatment can be postponed or elective. There are no significant differences in tumor size and lymph node metastasis between patients with delayed treatment ≤180 days and those without delayed treatment (41). However, a timely, safe, protective environment is recommended for patients with aggressive lymph node metastasis or suspected extrathyroidal metastasis (42). These aggressive tumors can rapidly progress to metastatic disease or death (43).

Preoperative considerations must be more comprehensive in a pandemic situation (44). Pulmonary function should be thoroughly evaluated in a multidisciplinary environment. Bronchoalveolar lavage is recommended during bacterial culture and antibiotic susceptibility testing to exclude residual SARS-CoV-2 infection (45). However, if surgery cannot be performed within an acceptable time frame, alternative treatments (e.g., RAI or pharmacotherapy) should be considered (46).

### RAI Treatment

^131^I treatment is a vital tool for thyroid cancer treatment; it involves the irradiation of residual thyroid or unresectable/incompletely resected thyroid cancer (47). For patients with advanced DTC, distant metastasis, or apparent extrathyroidal invasion, ^131^I treatment is the main first-line option. ^131^I treatment increases Treg count and decreases B lymphocyte count, leading to an immune imbalance (48). However, there has been no clinical confirmation of immunosuppressive status, implying that the clinical effects of toxicity are limited (49). ^131^I treatment does not enhance the risk, morbidity, or mortality of COVID-19 (50). Thus, ^131^I treatment should not be delayed in patients with suspected distant metastases.

## Multikinase Inhibitors (MKIs) for Thyroid Cancer During COVID-19 Pandemic

Although the treatment of DTC has potential for good long-term survival, advanced DTCs and ATCs have poor prognoses because of distant metastasis or resistance to the standard treatment options. Targeted therapy has been a new therapeutic method; new drugs are also emerging. Thyroid cancer has high expression levels of VEGF and its receptor (VEGFR), as well as frequent mutations in genes such as RET, BRAF, and RAS. The phosphoinositide 3-kinase/Akt/mTOR and mitogen-activated protein kinase signaling pathways have critical roles in thyroid cancer; several MKIs block tyrosine kinase receptors, thus preventing angiogenesis and tumor growth through these two pathways (51). MKIs acting on these targets can prolong median progression-free survival (PFS) and reduce tumor size (52, 53) **(** **Table 2** **).**

**Table 2:** Multikinase inhibitors for thyroid cancer during COVID-19 pandemic.

Drug trade name	Drug code	Primary targets	FDA-approved therapeutic indications	Clinical trials applied in thyroid cancer	Clinical trials	Relationship to COVID-19 treatment
**Anlotinib**	AL3818	VEGFR1–3 PDGFR	NSCLC	MTC: 57%PR (54, 55)	phase I clinical trials	–
		FGFR1/2			phase II clinical trials	
**Axitinib**	AG-013736	VEGFR1-3	RCC	DTC: 6%CR, 24%PR	phase II clinical trials	–
				MTC: 27%PR (56, 57)		
**Cabozantinib**	BMS-907351	VEGFR2, MET, RET	MTC, RCC, HCC	MTC: 68%PR, 41%SD ≥6 months	phase I clinical trials	experts recommend that people without obvious contraindications continue to use in the treatment of advanced renal cell carcinoma (58)
				DTC: 53%PR (59–62)	phase II clinical trials	
					phase III clinical trials	
**Dabrafenib combination with Trametinib**	GSK2118436	Dabrafenib: BRAF V600E	BRAF-mutated ATC, melanoma, NSCLC	ATC: 63%overall response 60weeks PSF (63)	phase II clinical trials	bind tightly to 6lu7 of SARS-CoV-2, inhibit the replication of SARS-CoV-2 (64–66)
	GSK1120212	Trametinib: MAPK				
**Dovitinib**	TKI258	VEGFR	metastatic NSLCL	DTC: 21%PR	phase II clinical trials	–
		FGFR		MTC: 17%PR (67)		
		PDGFR				
**Entrectinib**	RXDX-101	NTPK, BRAF	solid tumors with NTRK fusion proteins, ROS1-positive NSCLC	ROS1-fused DTC: peri-aortic nodules, liver metastases disappeared (68)	phase II clinical trials	antiviral activity against SARS-CoV-2 in human lung tissue (69, 70)
		ALK, ROS1				
**Everolimus**	RAD001	mTOR	HER2(-) breast cancers, pancreatic, neuroendocrine tumors, RCC, angiomyolipomas, subependymal giant cell astrocytomas	12.9 months PSF	phase II clinical trials	reduce conventional T lymphocyte proliferation, attenuate cytokine storm, maintain regulatory T cell growth and activity (71, 72)
				DTC: 3% PR		
				MTC: 10% PR		
				ATC: 14% PR (73, 74)		
**Larotrectinib**	LOXO-101	NTRK gene fusions	solid tumors with NTRK fusion proteins	PTC: iodine uptake was reactivated after three weeks (75)	phase I clinical trials	through high-throughput virtual screening and docking mode, potential drug candidates of COVID-19 (76)
**Lenvatinib**	AK175809	VEGFR1–3	DTC, RCC (combination with Everolimus)	DTC: 64.8%(PR+CR) 18.3months PSF	phase II clinical trials, phase III clinical trials	significant synergy with remdesivir to inhibit SARS-CoV-2 replication (77, 78)
		FGFR1–4		MTC: 36%PR, 44%SD, 9 months PSF		
		PDGFR, RET		ATC (combination with pembrolizumab): 66%CR, 16%SD,16%PD, 16.5months PSF (53, 79–83)		
**Pazopanib**	GW786034	VEGFR1–3 PDGFR	RCC, soft tissue sarcomas	DTC:49% PR	phase II clinical trials	may disrupt the binding of SARS-CoV-2 to csBiP, may be drug candidates for COVID-19 (84)
		FGFR1/2		MTC: 57% SD, 9.4 months PSF (85–87)		
**Pralsetinib**	BLU-667	RET	RET fusion-positive NSCLC	MTC:4% CR, 36% PR	phase I clinical trials	_
				PTC (1): achieved PR, the tumor shrunk by 70% (88)		
**Selpercatinib**	LOXO-292	RET	RET-mutated MTC, RET fusion-positive NSCLC	MTC: with extensive metastases and inoperable, after selpercatinib was administered, surgery was performed (89)	neoadjuvant therapy	bind strongly to four isolated SARS-CoV-2 proteins (90)
**Sorafenib**	BAY 43-9006	VEGFR1–3 PDGFR, RET	DTC, RCC, HCC	DTC:23%PR,53%SD, 79 weeks PSF	animal experiment	may be associated with differentially expressed genes identified in SARS-CoV-2 infection (77, 91, 92)
				MTC: 47% PR, 40% SD (52, 93–97)	phase II clinical trials	
					phase III clinical trials	
**Sunitinib**	SU11248	VEGFR1–3 PDGFR, RET	GIST, pancreatic neuroendocrine tumors, RCC	DTC: response (22%)	phase II clinical trials	effective antiviral properties against SARS-CoV-2 (98, 99)
				ATC:38%PR (100, 101)		
**Vandetanib**	ZD6474	VEGFR2/3 EGFR, RET	MTC	MTC:44%PR, 30.5 months PSF	phase II clinical trials	reduce inflammatory cytokines and pulmonary infiltration in animal models of SARS-CoV-2 infection and A549-ACE2 cells (102)
				DTC:8.3%PR,11.3 months PSF (103–109)	phase III clinical trials	
**Vemurafenib**	PLX-4032	BRAF V600E	BRAF V600E melanomas	PTC: 38.5% PR, 57.5% SD,18.2 months PFS (110)	phase II clinical trials	may hinder viral attachment and replication by locking the SBD in a closed conformation triggering apoptosis in infected cells (84, 111, 112)

(Clinical trials applied in thyroid cancer: choose the best experimental results).

ALK, anaplastic lymphoma kinase; ATC, anaplastic thyroid cancer; BRAF, B-raf proto-oncogene, serine/threonine kinase; DTC, differentiated thyroid cancer; EGFR, epidermal growth factor receptor; GIST, gastrointestinal stromal tumors; HCC, hepatocellular carcinomas; HER2, human epidermal growth factor receptor-2; MAPK, mitogen-activated protein kinases; MTC, medullary thyroid carcinoma; mTOR, mechanistic target of rapamycin; NTRK, neurotrophic tropomyosin receptor kinase; NSCLC, non-small cell lung cancers; PDGFR, platelet-derived growth factor; RET, rearrangement during transfection; RCC, renal cell carcinomas;ROS1, ros oncogene1 kinase; VEGFR, vascular endothelial growth factor.

### FDA-Approved MKIs for Thyroid Cancer

#### Vandetanib

Vandetanib is the first FDA-approved drug for the treatment of advanced MTC. Vandetanib selectively blocks epidermal growth factor receptor, VEGFR, and RET-tyrosine kinase (113). Hereditary MTCs and half of sporadic MTCs have RET mutations (114). Vandetanib can inhibit tumor cell proliferation in a wide range of preclinical models and phase II clinical trials (103). Wells et al. found that in MTC patients who received vandetanib treatment, 20% had partial remission (PR), while 53% had stable disease (SD) for more than 24 weeks (104). In another cohort of MTC patients, Robinson et al. found that 16% had PR and 53% had SD for at least 24 weeks after vandetanib treatment (105). In a phase III clinical trial of vandetanib for the treatment of MTC, Wells et al. found PR in 44% of patients and a median PFS of 30.5 months (106). Additionally, vandetanib was effective in adolescents with hereditary MTC and ectopic Cushing’s syndrome caused by progressive MTC (107, 108). In a phase II clinical trial, Leboulleux et al. found that vandetanib prolonged the median PFS to 11.1 months and led to PR in 8.3% of patients with advanced RAI-refractory DTC (109).

COVID-19 is often associated with a cytokine storm response involving inflammatory cytokines, which leads to multiple organ dysfunction. Importantly, MKIs have anti-inflammatory and cytokine inhibitory activities. They may be used for broader spectrum antiviral therapy and can reduce the possibility of life-threatening diseases caused by respiratory virus infection-related lung injury (115). MKIs may be useful as treatment for COVID-19 (115). Puhl et al. evaluated the efficacy of vandetanib in an animal model and A549–angiotensin-converting enzyme 2 cells that had been infected with SARS-CoV-2. The treatment significantly reduced inflammatory cytokines and immune cell infiltration in the lungs, suggesting that vandetanib can be used for treatment of COVID-19 (102).

#### Cabozantinib

Cabozantinib blocks VEGFR, c-MET, and RET; it is the second FDA-approved drug for the treatment of progressive and symptomatic MTC (116). Compared to vandetanib, cabozantinib has more potent antiangiogenic activity (117). Bentzien et al. demonstrated that cabozantinib effectively inhibited the growth of MTC tumors (59). In a phase I study of cabozantinib for the treatment of advanced MTC, 41% of patients had SD for 6 months, while 68% of patients had confirmed PR (60). The selection of vandetanib or cabozantinib is made according to the patient’s age, comorbidities, disease manifestations, and progression. Thus far, clinical evidence suggests that vandetanib is better tolerated, compared to cabozantinib. Patients with a poor constitution and older patients may prefer vandetanib. However, patients receiving cabozantinib may experience few or no side effects (61). Similar to vandetanib, cabozantinib has not yet been approved for the treatment of DTC. However, in a study of 15 patients with DTC who had not responded to standard RAI therapy, Cabanillas et al. found that eight patients (53%) experienced PR after treatment with cabozantinib (62).

The COVID-19 pandemic has required more careful selection of targeted drugs. Experts recommend continued use of cabozantinib in patients without obvious contraindications (58). Thus far, there remains no relevant research concerning the use of cabozantinib in the treatment of thyroid cancer.

#### Sorafenib

Sorafenib is approved for the treatment of RAI-refractory DTC. It blocks VEGFR, platelet-derived growth factor, fibroblast growth factor, and RET receptors (118). Three prospective trials had shown that sorafenib was effective against metastatic thyroid cancer, including PTC and RAI-refractory DTC (52, 93, 94). In a clinical trial of sorafenib by Abramson et al., 23% of patients had PR and 53% of patients had SD; the median PFS was 79 weeks (93). Additional studies had shown that sorafenib was effective for the treatment of MTC (95). Sorafenib had also been shown to inhibit the growth of orthotopic ATC xenografts and improve survival in experimental animals with ATC (96). However, Ito et al. reported that sorafenib was not effective for treating ATC (97).

Sorafenib has been reported to exhibit antiviral effects. It prevents viral egress by Rift Valley fever virus through the inhibition of valosin-containing protein function (91). A new computational approach has suggested that sorafenib treatment interacts with genes that are differentially expressed during SARS-CoV-2 infection, offering a potential therapeutic candidate for COVID-19 (92). Additionally, a drug interaction has been reported between MKIs and medications used for COVID-19 treatment; the interaction causes QT prolongation and CYP3A4 inhibition. Therefore, sorafenib should be discontinued during chloroquine/hydroxychloroquine treatment. If symptoms such as hypokalemia and fever occur, MKIs should be stopped immediately and a cardiology consultation should be conducted (77).

Similarly, lopinavir/ritonavir should be used with caution in patients receiving sorafenib. Patients with thyroid cancer who received MKIs reportedly experienced more COVID-19 virus infections, compared to patients who did not receive MKIs (119). Among patients with thyroid cancer, a greater risk of COVID-19 complications is associated with MKI treatment or external beam radiation therapy, the presence of lung metastases, older age, and the presence of other comorbidities (37).

#### Lenvatinib

Similar to other MKIs, lenvatinib exerts its actions by targeting tumor angiogenesis (120). It is mainly approved for RAI-refractory DTC treatment. Tohyama et al. reported that lenvatinib exhibited significant antitumor and antiangiogenic activity in a DTC xenograft model (79). Schlumberger et al. found that the median PFS was 18.3 months, and the response rate was 64.8% in the lenvatinib group (53). In contrast, Brose et al. conducted a phase III trial concerning the effect of age (≤65 or >65 years) on the efficacy of lenvatinib in patients with RAI-refractory DTC. The results showed a median PFS of 20.2 vs. 3.2 months and 16.7 vs. 3.7 months; elderly patients treated with lenvatinib had better overall survival. In contrast, younger patients had a significantly higher objective response rate than did older patients (72% vs. 55%) (80). Lenvatinib is also effective for the treatment of MTC. In a preclinical study conducted by Tohyama et al., lenvatinib showed significant antitumor activity in a model of MTC (79). In a phase II trial of lenvatinib for the treatment of advanced MTC, Schlumberger et al. showed that 36% of patients had PR, while 44% of patients had SD; the median PFS was 9 months (81). There is limited available data regarding lenvatinib for the treatment of ATC. In a preclinical study performed by Tohyama et al., lenvatinib had significant antitumor and antiangiogenic activity in an ATC xenograft model (79). However, Wirth et al. reported that lenvatinib monotherapy may not be an effective treatment for ATC (82). In a trial of lenvatinib combined with pembrolizumab for the treatment of ATC, Dierks et al. showed that 66% of patients had CR, while 16% had SD and 16% had PD; the median PFS was 16.5 months (83).

Pohl et al. (78) screened 6000 compounds within the DrugBank library for their potential to inhibit the SARS-CoV-2 3CL (i.e., 3C-like) main protease *via* high-throughput docking. They found that lenvatinib and remdesivir have obvious synergistic effects, which can inhibit SARS-CoV-2 replication. They validated this finding with cellular experiments; importantly, this synergistic inhibition was not caused by blocking interactions with VEGFR or platelet-derived growth factor receptor. The synergistic effect may be related to more efficient inhibition of viral transcription and replication processes, rather than any effect on early stages of the viral replication cycle. Locantore et al. reported the first case of a lenvatinib-treated thyroid cancer patient who developed COVID-19. The patient was diagnosed with DTC and had bilateral lung metastases; the patient was administered lenvatinib and exhibited partial response to treatment, along with reduction of lung metastases. Subsequently, the patient had a positive SARS-CoV-2 test result during treatment; the disease was initially asymptomatic, then progressed to cough and diarrhea after a few days. However, it did not progress to anosmia or fever; the patient continued lenvatinib and underwent daily monitoring of vital signs. Twenty-one days later, SARS-CoV-2 test results were negative, and no additional adverse events occurred (77). These findings suggest that continued lenvatinib treatment is safe during the COVID-19 pandemic, but there remains a need to monitor the safety profile and associated complications of continued cancer treatment in COVID-19 patients through larger studies and longer follow-up periods.

#### Dabrafenib and Trametinib

Dabrafenib is a selective BRAFV600E-mutant kinase inhibitor, and trametinib is a mitogen-activated protein kinase inhibitor. The FDA has approved the two drugs for combination therapy for ATC with the BRAFV600E mutation (121). Subbiah et al. reported a 63% overall response rate and median PFS of 60 weeks in patients who had ATC with the BRAFV600E mutation (63). Islam et al. analyzed publicly available transcriptomic RNA-Seq data from patients with COVID-19 to identify differentially expressed genes using bioinformatics and network biology; they also performed drug retargeting analysis of hub genes. The computer analysis showed that dabrafenib was validated as the highest-scoring repurposed drug, suggesting its use as a potential treatment for COVID-19 (64). Through cell-based experiments, Wan et al. confirmed that dabrafenib could inhibit SARS-CoV-2 replication for potential treatment of COVID-19 (65). Chtita et al. performed virtual screening of approved drugs, then used virtual screening and molecular docking studies to evaluate the active site of the major protease (6lu7) of SARS-CoV-2; they showed that trametinib binds tightly to 6lu7, indicating that this drug may be effective for the treatment of COVID-19 (66).

#### Selpercatinib

Selpercatinib is a selective RET receptor inhibitor approved by the FDA for the treatment of RET-mutated MTC (122). Jozaghi et al. reported the use of neoadjuvant therapy with selpercatinib in a patient who had RET-mutated MTC with extensive metastases (89). Al-Zaqri et al. evaluated the structural and electronic characteristics of selpercatinib through computational studies, various quantum mechanical analyses, and docking studies. They found that selpercatinib strongly binds to four isolated SARS-CoV-2 proteins; the results suggested that selpercatinib may mimic the activity of a SARS-CoV-2 protein, thus blocking SARS-CoV-2 protein function and offering a potential treatment for COVID-19 (90).

In addition to these FDA-approved kinase inhibitors for the treatment of thyroid cancer, other kinase inhibitors are used for clinical treatment of thyroid cancer.

### Neurotrophic Tyrosine Receptor Kinase (NTRK) Inhibitors

#### Entrectinib

Entrectinib is a novel, potent inhibitor of anaplastic lymphoma kinase, c-ros oncogene 1 kinase **(**ROS1), and important NTRK that treats tumors harboring oncogenic forms of these proteins (123). Liu et al. reported the use of entrectinib in a patient with ROS1-fused DTC who had DTC with metastases; they showed favorable treatment outcomes with peri-aortic nodules and the disappearance of liver metastases (68). El-aarag et al. used bioinformatics analysis to identify differentially expressed genes related to COVID-19, then used the Drug-Gene Interaction Database to search for promising drugs; they suggested that entrectinib could serve as a novel treatment for COVID-19 (69). Peralta-Garcia et al. reported the antiviral activity of entrectinib against SARS-CoV-2 in human lung tissue and in an antiviral assay involving human lung tissue cells (70).

#### Larotrectinib

Larotrectinib is a selective NTRK inhibitor approved by the FDA for the treatment of solid tumors with NTRK gene fusions (124). Groussin et al. described a patient with PTC and lung metastases who did not respond to RAI treatment. That patient’s PTC exhibited a fusion gene (EML4-NTRK3) and iodine uptake was reactivated after 3 weeks of larotrectinib therapy, suggesting that retreatment with RAI should be considered in patients receiving larotrectinib (75). Abhithaj et al. identified larotrectinib as a potential drug candidate for the treatment of COVID-19 through high-throughput virtual screening, as well as ultra-precision and standard precision docking models (76).

### BRAF Inhibitors

Vemurafenib is an inhibitor of the mutant serine-threonine kinase BRAF; it potently inhibits ERK phosphorylation and cell proliferation only in BRAF mutant cell lines (125). In a phase II trial of vemurafenib for the treatment of BRAFV600E-mutated PTC, Brose et al. showed that 38.5% of patients had PR and 57.5% of patients had SD; the median PFS was 18.2 months (110). Cell surface-bound immunoglobulins include a substrate-binding domain (SBD) that binds to polypeptides and a nucleotide-binding domain (111) that initiates exogenous caspase-dependent apoptosis. In contrast, cell surface-bound immunoglobulins can bind to SARS-CoV-2 (112). Zhang et al. used *in silico* analyses to identify 10 nucleotide-binding domain-binding candidates, including vemurafenib and nucleotide-binding domain-binding drugs that may hinder viral attachment and replication by locking the SBD in a closed conformation that triggers apoptosis in infected cells; the findings suggest that vemurafenib can be used for the treatment of COVID-19 (84).

### Antiangiogenic Inhibitors

Angiogenesis is the primary pathogenesis pathway in thyroid cancer. Some antiangiogenic MKIs (e.g., pazopanib, sunitinib, anlotinib, axitinib, and dovitinib) are effective for the treatment of thyroid cancer, mainly through the inhibition of VEGFR, fibroblast growth factor 1/2, platelet-derived growth factor receptor, and RET receptors (126). In a phase II clinical trial, Bible et al. showed that pazopanib has a therapeutic effect in patients with DTC or MTC (85, 86). They also conducted clinical trials involving patients with ATC, but the drug showed minimal activity in such patients (87). In a phase II clinical trial, Carrd et al. showed that sunitinib was effective for the treatment of DTC and MTC (100). Ravaud et al. also showed that sunitinib was effective for the clinical treatment of DTC but not ATC (101). Anlotinib has encouraging efficacy and a manageable and tolerable safety profile in many malignancies, including MTC (54). Sun et al. conducted a phase II clinical trial to confirm the antitumor activity of anlotinib in patients with advanced or metastatic MTC; they reported PR in 57% of patients (55). Capdevila et al. performed clinical trials of treatments for advanced RAI-resistant DTC and refractory MTC. They found better outcomes with first-line axitinib; the objective response rate was 53% and the median PFS was 13.6 months (56). Cohen showed that axitinib had significant antitumor activity in all histological subtypes of advanced thyroid cancer (57). Lim et al. demonstrated moderate activity and manageable toxicity of dovitinib in the treatment of locally advanced or metastatic TC, with PR in 21% of patients with DTC and 17% of patients with MTC (67).

During the COVID-19 pandemic, there have been attempts to identify the potential effects of these drugs on patients with COVID-19. Kinase inhibitors have clear roles in reducing the infectivities of viruses such as Ebola and hepatitis C, suggesting that drugs such as sunitinib may be effective against SARS-CoV-2 (98). Girgis et al. performed chemical modifications of sunitinib, then verified the antiviral properties of the products against SARS-CoV-2; they reported that sunitinib may be effective for the treatment of COVID-19 (99). Zhang et al. identified 10 SBD-binding drug candidates (including pazopanib); the predicted SBD-binding drugs may disrupt SARS-CoV-2 binding interactions with cell surface-bound immunoglobulins, suggesting that pazopanib can be used for the treatment of COVID-19 (84).

### RET Inhibitors

Pralsetinib is a selective RET receptor inhibitor approved by the FDA for the treatment of RET fusion-positive non-small cell lung cancer (127). Subbiah et al. found that among MTC patients, 4% had CR and 36% had PR, while the single PTC patient in the study also achieved PR and the tumor shrunk by 70%; the results suggest that pralsetinib is suitable for the treatment of RET-altered TC (88).

### Serine-Threonine Kinase Inhibitors of mTOR

Everolimus is an inhibitor of the mTOR serine/threonine kinase signal transduction pathway (128). The FDA has approved it for the treatment of HER2(-) breast cancers, pancreatic neuroendocrine tumors, and angiomyolipomas (129). Lim et al. conducted a phase II trial of everolimus in locally advanced or metastatic thyroid cancer; the results showed limited activity and low response rates (73). Hanna et al. found PR in 3% of patients with DTC, 10% of patients with MTC, and 14% of patients with ATC; the median PFS was 12.9 months (74).

Everolimus-mediated mTOR inhibition may help to manage COVID-19 by reducing conventional T lymphocyte proliferation, attenuating the cytokine storm phenomenon, and maintaining regulatory T cell growth and activity (71). However, the dosage, safety, and adverse reactions of everolimus for the treatment of COVID-19 require further research (72).

## Immunotherapy

The development and approval of cancer-specific immunotherapy has led to new avenues and methods for the treatment of malignant tumors (130). Thus far, few immunotherapy trials have focused on TC. Pembrolizumab is a humanized monoclonal anti-PD-1 antibody approved by the FDA for the treatment of advanced melanoma and NSCLC (131). Mehnert et al. conducted a clinical trial of pembrolizumab in patients with advanced PD-L1-positive DTC. The results showed that the median overall survival was not reached, while two patients (9%) achieved PR, indicating that pembrolizumab has antitumor activity in a few patients with advanced DTC (132). Spartalizumab is an immunoglobulin-4 monoclonal antibody that binds PD-1, thereby blocking its interactions with PD-L1 and PD-L2 (133). Capdevila et al. conducted a clinical trial of the therapeutic effect of spartalizumab in ATC. The results showed that 7% of patients had CR, while 12% of patients had CR; the 1-year survival rate in the PD-L1-positive population was 52.1%. These findings indicate that spartalizumab has a therapeutic effect in patients with PD-L1-positive ATC (134). Loretelli et al. analyzed the immunological characteristics of patients who recovered from COVID-19; they found that PD-1 blockade normalized the immune phenotype and restored T-cell function, thereby improving immune abnormalities after COVID-19. This treatment approach can also stimulate an anti-SARS-CoV-2 immune response (135). Thus, pembrolizumab and spartalizumab may be effective for the treatment of COVID-19.

## Conclusion

The impact of COVID-19 on thyroid cancer is unprecedented. It affects the diagnosis, treatment, and management of thyroid cancer. Diagnosis is delayed and treatment requires more careful choices. However, in the case of the gradual normalization of the epidemic situation, the treatment of thyroid cancer should continue. Compared to surgery and RAI treatment, drug therapy appears to be a safer option. Some targeted therapies (e.g., MKIs and immunotherapy) have demonstrated robust effects on thyroid cancer, and some drugs are also effective in treating COVID-19. If appropriate drugs and safe doses can be selected, it will be better for patients with thyroid cancer combined with COVID-19. However, the drug dosage and safety must be validated in future studies.

## Author Contributions

FH and XS: Conceptualization, Writing - Review and Editing. NQ, ZH, and ZS: Methodology, Software, Visualization, Writing - Original Draft. CK and NH: Visualization, Writing- Review. All authors contributed to the article and approved the submitted version.

## Funding

This study was supported by grants from National Natural Science Foundation of China (81870593, 82170865), Natural Science Foundation of Shandong Province (ZR2020MH106), Shandong Province Higher Educational Science and Technology Program for Youth Innovation (2020KJL004), and Doctor Startup Fund of Affiliated Hospital of Weifang Medical University (2020BSQD01).

## Conflict of Interest

The authors declare that the research was conducted in the absence of any commercial or financial relationships that could be construed as a potential conflict of interest.

## Publisher’s Note

All claims expressed in this article are solely those of the authors and do not necessarily represent those of their affiliated organizations, or those of the publisher, the editors and the reviewers. Any product that may be evaluated in this article, or claim that may be made by its manufacturer, is not guaranteed or endorsed by the publisher.
